# Prediction of Long-Term Treatment Response to Selective Serotonin Reuptake Inhibitors (SSRIs) Using Scalp and Source Loudness Dependence of Auditory Evoked Potentials (LDAEP) Analysis in Patients with Major Depressive Disorder

**DOI:** 10.3390/ijms16036251

**Published:** 2015-03-18

**Authors:** Bun-Hee Lee, Young-Min Park, Seung-Hwan Lee, Miseon Shim

**Affiliations:** 1 Department of Psychiatry, Seoul Eunpyeong Hospital, 90, Baengnyeonsan-ro, Eunpyeong-gu, Seoul 122-913, Korea; E-Mail: punzza91@naver.com; 2 Department of Psychiatry, Ilsan Paik Hospital, Inje University College of Medicine, 2240, Daehwa-dong, Ilsanseo-gu, Goyang 411-706, Korea; E-Mail: lshpss@hanmail.net; 3 Clinical Emotion and Cognition Research Laboratory, Inje University, Goyang 411-706, Korea; E-Mail: miseon@bme.hanyang.ac.kr; 4 Department of Biomedical Engineering, Hanyang University, Seoul 133-791, Korea

**Keywords:** antidepressants, loudness dependence of auditory evoked potentials (LDAEP), standardized low resolution brain electromagnetic tomography (sLORETA), major depressive disorder, response, serotonin

## Abstract

Background: Animal and clinical studies have demonstrated that the loudness dependence of auditory evoked potentials (LDAEP) is inversely related to central serotonergic activity, with a high LDAEP reflecting weak serotonergic neurotransmission and *vice versa*, though the findings in humans have been less consistent. In addition, a high pretreatment LDAEP appears to predict a favorable response to antidepressant treatments that augment the actions of serotonin. The aim of this study was to test whether the baseline LDAEP is correlated with response to long-term maintenance treatment in patients with major depressive disorder (MDD). Methods: Scalp N1, P2 and N1/P2 LDAEP and standardized low resolution brain electromagnetic tomography-localized N1, P2, and N1/P2 LDAEP were evaluated in 41 MDD patients before and after they received antidepressant treatment (escitalopram (*n* = 32, 10.0 ± 4.0 mg/day), sertraline (*n* = 7, 78.6 ± 26.7 mg/day), and paroxetine controlled-release formulation (*n* = 2, 18.8 ± 8.8 mg/day)) for more than 12 weeks. A treatment response was defined as a reduction in the Beck Depression Inventory (BDI) score of >50% between baseline and follow-up. Results: The responders had higher baseline scalp P2 and N1/P2 LDAEP than nonresponders (*p* = 0.017; *p* = 0.036). In addition, changes in total BDI score between baseline and follow-up were larger in subjects with a high baseline N1/P2 LDAEP than those with a low baseline N1/P2 LDAEP (*p* = 0.009). There were significantly more responders in the high-LDAEP group than in the low-LDAEP group (*p* = 0.041). Conclusions: The findings of this study reveal that a high baseline LDAEP is associated with a clinical response to long-term antidepressant treatment.

## 1. Introduction

Major depressive disorder (MDD) is a common psychiatric disorder. MDD is a condition characterized by single or recurrent major depressive episodes with personal suffering and significant social and functional impairment [1]. Many antidepressant agents have been introduced and used for treating MDD, but remission rates for MDD remain low [2,3,4]. To improve treatment efficiency for MDD, many investigators have tried to find a marker to predict a response to antidepressant treatment [5,6,7,8,9]. Although the findings have been controversial, they have proposed various parameters of genetics, proteomics, metabolics, neuroendocrinology, neuroimaging, and neurophysiology as potential (if not promising) candidate markers to predict a treatment response [6]. Among them, it has been suggested that the event-related potentials from an electroencephalogram (EEG) could be a useful marker to predict a antidepressant response because it is noninvasive and easy to apply and can measure central serotonergic activity [10,11].

For some time, the loudness dependence of auditory evoked potentials (LDAEP), which is measured using the event-related potentials associated with auditory processing, has been used as a noninvasive method for measuring central serotonergic activity [12]. Some animal and clinical studies have revealed that the LDAEP is a reliable marker of central serotonergic activity in psychiatric disorders including MDD [13,14,15]. It has been shown that the LDAEP is inversely related to central serotonergic activity, with a high LDAEP reflecting weak serotonergic neurotransmission and *vice versa* [16]. However, the animal studies consistently demonstrated such an inverse relationship between LDAEP and central serotonergic activity, while the studies in human showed less consistent findings. In animal studies, the microinjection into the dorsal raphe nucleus or systemic administration of a serotonin agonist or antagonist led to a decrease or increase, respectively, in the intensity dependence of the auditory evoked potential (AEP) [13,14,17]. However, research on humans examining AEP after treatment with tryptophan depletion, which reduces central serotonin levels, yielded variable results in intensity-dependent N1/P2 amplitudes or LDAEP slopes, including unaltered [18,19], increased [20], and decreased values [21,22]. Moreover, some studies, although few, have explored the relationship between LDAEP and other neurotransmitters, including dopamine and glutamate, though findings are insufficient to reach conclusions about their relationship [13,23]. One recent review has concluded that the LDAEP has a lack of sensitivity and specificity to acute changes in serotonergic neurotransmission, but that LDAEP can be a potential predictor of antidepressant treatment response [10].

Several clinical studies have examined the relationship between the LDAEP and response to antidepressant treatment in MDD [24]. An earlier study found that MDD patients with a high pretreatment LDAEP had a significantly greater amelioration of their depressive symptoms after four weeks of treatment with a selective serotonin reuptake inhibitor (SSRI) compared to those with a low pretreatment LDAEP [25]. This suggests that MDD patients with low serotonergic activity had a more favorable response to the serotonin agonist than those with high serotonergic activity. Similar results have been reported in subsequent studies [16,26,27]. In addition, most of the relevant studies have demonstrated a response to four weeks of acute treatment. However, a recent study did not find anything with baseline scalp LDAEP, though it did with baseline source LDAEP analysis for long-term treatment (12 weeks) [28].

The aim of the present study was to test the hypothesis that the pretreatment cortical and source LDAEP in MDD patients is correlated with the response to long-term maintenance treatment (*i.e.*, more than 12 weeks) with SSRIs. The LDAEP was examined in MDD patients before and after they were treated with SSRIs. The pre- and post-treatment LDAEP values were compared between those who responded to the long-term treatment (*i.e*., responders) and those who did not (nonresponders). In addition, changes in the severity of depression over the treatment period were compared between MDD patients with high and low pretreatment LDAEP.

## 2. Results and Discussion

The total Beck Depression Inventory (BDI) score for the entire cohort decreased significantly between baseline and follow-up (*t* = 6.981, *p* < 0.01), but there were no significant changes in the N1, P2, and N1/P2 LDAEP at the Cz electrode (*t* = 1.265, degree of freedom (df) = 40, *p* = 0.213; *t* = 0.617, df = 40, *p* = 0.541; *t* = −0.548, df = 40, *p* = 0.587, respectively) from baseline to follow-up. Table 1 presents the comparison of pretreatment and post-treatment LDAEP between groups according to sex, the number of episodes, hypnotic medication, and smoking. There were no significant differences in the baseline N1, P2, and N1/P2 LDAEP between males and females. The baseline N1, P2, and N1/P2 LDAEP had no significant differences between first- and recurrent-episode MDD patients. In addition, the pre- and post-treatment LDAEP values did not differ significantly between subjects with and without hypnotic medication or between those who did or did not smoke.

### 2.1. Responders vs. Nonresponders; Remitters vs. Nonremitters

**Figure 1:**
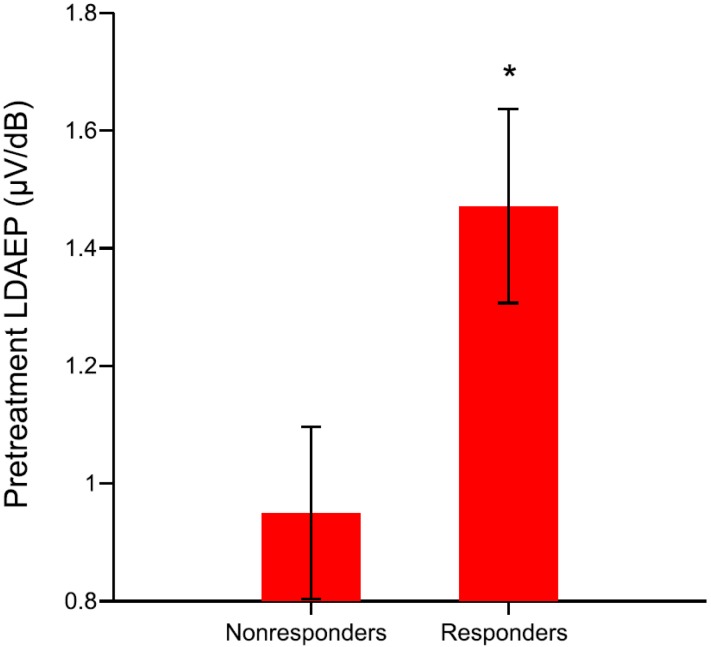
The pretreatment N1/P2 loudness dependence of auditory evoked potentials (LDAEP) of responders and nonresponders (responders were defined as those with a reduction in the Beck Depression Inventory (BDI) score of >50% between baseline and follow-up) among major depressive disorder (MDD) patients (*t* = −2.176, *p* = 0.036). The data are presented as mean and standard error values. * Statistically significant difference at *p* < 0.05.

The demographic data, clinical variables, and LDAEP are presented according to treatment response in Table 2. The pretreatment N1 LDAEP, N1 standardized low resolution brain electromagnetic tomography (sLORETA)-LDAEP, P2 sLORETA-LDAEP, and N1/P2 sLORETA-LDAEP did not significantly differ between the responders and nonresponders (*t* = 0.249, df = 39, *p* = 0.805; *t* = 0.155, df = 39, *p* = 0.878; *t* = −0.611, df = 39, *p* = 0.545; *t* = 0.822, df = 39, *p* = 0.922, respectively; Table 2). However, the responders had higher pretreatment cortical P2 and N1/P2 LDAEP than nonresponders (*t* = −2.498, df = 37.02, *p* = 0.017; *t* = −2.176, df = 39, *p* = 0.036, respectively; Figure 1). In addition, those LDAEP values differed significantly between the responders and nonresponders when sex was considered covariate in analysis (F(1, 40) = 4.105, *p* = 0.050; F(1, 40) = 4.198, *p* = 0.047, respectively).

**Table 1:** Comparison of pretreatment and post-treatment loudness dependence of auditory evoked potentials (LDAEP) between groups according to sex, the number of episodes, hypnotic medication, and smoking.

Sex	Male (*n* = 7)	Female (*n* = 34)	*p*
Pretreatment N1	−0.42 ± 0.70	−0.39 ± 0.50	0.919
P2	0.65 ± 0.63	0.92 ± 0.74	0.370
N1/P2	1.06 ± 1.09	1.31 ± 0.72	0.449
Post-treatment N1	−0.50 ± 0.56	−0.53 ± 0.58	0.909
P2	0.72 ± 1.11	0.81 ± 0.83	0.805
N1/P2	1.22 ± 1.10	1.34 ± 0.85	0.744
Recurrence	First-episode MDD (*n* = 15)	Recurrent-episode MDD (*n* = 26)	*p*
Pretreatment N1	−0.55 ± 0.66	−0.31 ± 0.42	0.174
P2	0.79 ± 0.68	0.92 ± 0.75	0.601
N1/P2	1.34 ± 0.89	1.23 ± 0.73	0.681
Post-treatment N1	−0.39 ± 0.74	−0.60 ± 0.45	0.344
P2	0.92 ± 0.83	0.72 ± 0.89	0.474
N1/P2	1.32 ± 0.97	1.32 ± 0.85	0.996
Hypnotics	No hypnotic medication (*n* = 20)	Hypnotic medication MDD (*n* = 21)	*p*
Pretreatment N1	−0.42 ± 0.62	−0.38 ± 0.43	0.827
P2	1.06 ± 0.75	0.69 ± 0.66	0.100
N1/P2	1.48 ± 0.91	1.07 ± 0.59	0.103
Post-treatment N1	−0.53 ± 0.56	−0.51 ± 0.59	0.912
P2	1.06 ± 1.01	0.55 ± 0.64	0.059
N1/P2	1.59 ± 0.94	1.06 ± 0.76	0.053
Smoking	No smoking (*n* = 31)	Smoking (*n* = 10)	*p*
Pretreatment N1	−0.46 ± 0.49	−0.20 ± 0.62	0.164
P2	0.90 ± 0.73	0.78 ± 0.70	0.649
N1/P2	1.37 ± 0.70	0.97 ± 0.97	0.171
Post-treatment N1	−0.49 ± 0.59	−0.63 ± 0.51	0.514
P2	0.88 ± 0.88	0.53 ± 0.81	0.268
N1/P2	1.37 ± 0.85	1.16 ± 1.01	0.509

Data are mean ± SD or *n* values; BDI, Beck depression inventory; LDAEP, loudness dependence of auditory evoked potentials.

**Table 2:** Comparison of demographic data, clinical variables, and LDAEP between nonresponder and responder groups among major depressive disorder (MDD) patients.

Variable	Nonresponders (*n* = 16)	Responders (*n* = 25)	*p*
Age (years)	43.0 ± 17.8	38.4 ± 13.4	0.387
Sex (male/female)	4/12	3/22	0.401
First-/Recurrent-episode	3/13	12/13	0.058
Nonsmoker/smoker	10/6	21/4	0.150
Pretreatment LDAEP (µV/dB)			
N1	−0.37 ± 0.53	−0.41 ± 0.53	0.805
P2	0.58 ± 0.40	1.06 ± 0.82	0.017 *
N1/P2	0.95 ± 0.59	1.47 ± 0.83	0.036 *
Post-treatment LDAEP (µV/dB)			
N1	−0.44 ± 0.35	−0.57 ± 0.67	0.419
P2	0.63 ± 0.73	0.90 ± 0.95	0.341
N1/P2	1.07 ± 0.73	1.47 ± 0.95	0.164
Baseline BDI score	28.9 ± 9.8	32.4 ± 13.8	0.394
Post-treatment BDI score	25.6 ± 9.5	5.5 ± 4.8	<0.01 **

Data are mean ± SD or *n* values; BDI, Beck depression inventory; LDAEP, loudness dependence of auditory evoked potentials; * Statistically significant difference at *p* < 0.05; ** Statistically significant difference at *p* < 0.01.

Although the pretreatment total BDI scores there did not differ between these two groups (*t* = −0.862, df = 39, *p* = 0.394), a significant difference was found in the post-treatment total BDI scores (*t* = 7.900, df = 19.91, *p* < 0.01). In addition, there were no significant changes in the N1, P2, and N1/P2 LDAEP (as assessed using the paired *t*-test) between baseline and follow-up in either the responders (*p* > 0.05) or nonresponders (*p* > 0.05).

**Figure 2:**
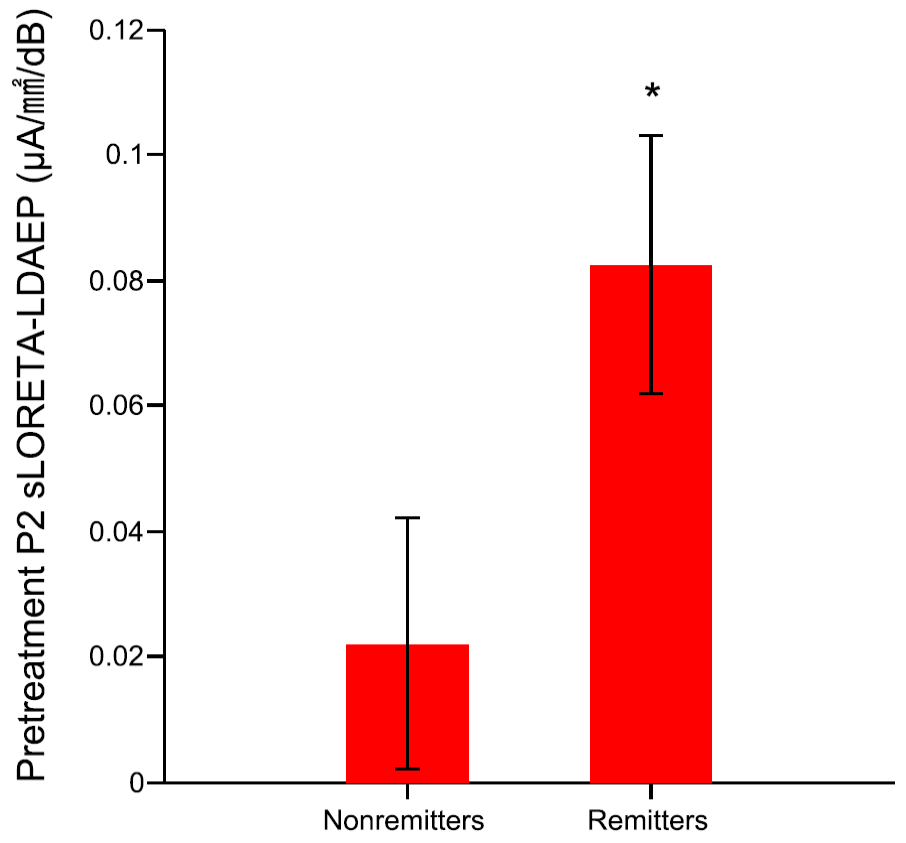
The pretreatment left P2 standardized low resolution brain electromagnetic tomography (sLORETA)-LDAEP of remitters and nonremitters (remitters were defined as those with <10 points in the post-treatment BDI score) among MDD patients (*t* = −2.095, *p* = 0.043). The data are presented as mean and standard error values. * Statistically significant difference at *p* < 0.05.

Furthermore, the remitters had higher pretreatment cortical P2 LDAEP and left P2 sLORETA-LDAEP than nonremitters (*t* = −2.095, df = 31.52, *p* = 0.044; *t* = −2.095, df = 39, *p* = 0.043, respectively; Figure 2).

### 2.2. Low vs. High Pretreatment Loudness Dependence of Auditory Evoked Potentials (LDAEP)

**Figure 3:**
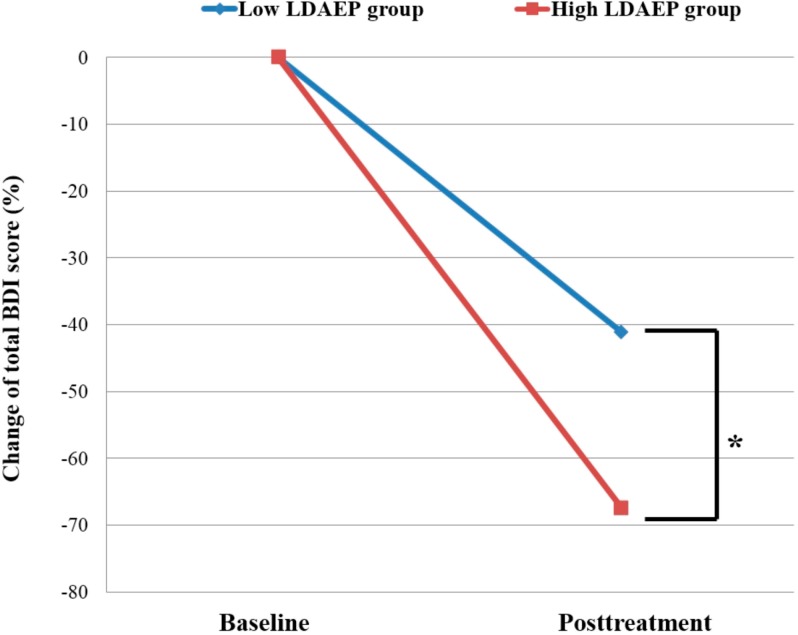
Changes in the total BDI score between baseline and follow-up in MDD patients dichotomized according to their pretreatment LDAEP (*i.e*., low or high). The change in total BDI score differed significantly between the low- and high-LDAEP groups (*t* = −2.741, *p* = 0.009). BDI change, change in BDI score from baseline to follow-up. * Statistically significant difference at *p* < 0.01.

Table 3 gives the characteristics of the patients dichotomized according to their pretreatment N1/P2 LDAEP. There was no significant difference in the pretreatment total BDI score between the low- and high-LDAEP groups (*t* = −1.260, df = 39, *p* = 0.215), while a significant difference was shown in the post-treatment total BDI scores (*t* = 2.47, df = 39, *p* = 0.018). Moreover, the change in BDI score between baseline and follow-up differed significantly between the two groups (*t* = −2.741, df = 39, *p* = 0.009; Figure 3). There was a significantly higher rate for responders in the high-LDAEP group (76.2%) than in the low-LDAEP group (45%) (χ^2^ = 4.188, *p* = 0.041). The odds ratio of baseline low N1/P2 LDAEP was 1.91 (95% confidential interval (CI), 1.02–3.58) and the odds ratio of high N1/P2 LDAEP was 0.49 (95% CI, 0.22–1.07) for treatment nonresponse. In addition, these showed the significant effect of BDI change differences between the baseline low- and high-LDAEP groups (Cohen’s d = 0.8).

**Table 3:** Comparison of demographic data, clinical variables, and the LDAEP between MDD patients with low and high pretreatment LDAEP (dichotomized at the median into low *vs.* high).

Variable	Low LDAEP Group (*n* = 20)	High LDAEP Group (*n* = 21)	*p*
Age (years)	44.2 ± 14.6	36.4 ± 15.1	0.102
Sex (male/female)	3/17	4/17	1.000
First-/Recurrent-episode	5/15	10/11	0.133
Nonsmoker/smoker	12/8	19/2	0.032 *
Pretreatment LDAEP			
N1	−0.18 ± 0.42	−0.61 ± 0.54	<0.01 **
P2	0.44 ± 0.44	1.28 ± 0.70	<0.01 **
N1/P2	0.62 ± 0.36	1.89 ± 0.52	<0.01 **
Post-treatment LDAEP			
N1	−0.35 ± 0.58	−0.68 ± 0.53	0.063
P2	0.39 ± 0.68	1.19 ± 0.86	<0.01 **
N1/P2	0.74 ± 0.68	1.87 ± 0.69	<0.01 **
Pretreatment BDI score	28.6 ± 10.6	33.4 ± 13.7	0.215
Post-treatment BDI score	17.9 ± 13.0	9.1 ± 9.6	0.018 *
BDI change (%)	37.2 ± 40.9	70.2 ± 36.2	0.009 **
Responder/nonresponder	9/11	16/5	0.041 *

Data are mean ± SD or *n* values; BDI, Beck depression inventory; BDI change, change of BDI score from baseline to post-treatment; LDAEP, loudness dependence of auditory evoked potentials; * Statistically significant difference at *p* < 0.05; ** Statistically significant difference at *p* < 0.01.

### 2.3. Discussion

The present study examined the relationship between the pretreatment LDAEP and the treatment response to antidepressant monotherapy in MDD patients who had received maintenance antidepressant medication for more than 12 weeks. The findings showed that the pretreatment scalp P2 and N1/P2 LDAEP was significantly higher in MDD patients with a treatment response (*i.e*., responders) than in those without a treatment response (*i.e*., nonresponders). Thus, the MDD treatment responders may have a tendency toward relatively low central serotonergic activity. Comparison of the low- and high-LDAEP groups (based on the median N1/P2 LDAEP) revealed that the latter included a higher proportion of responders. Also, when considering the odds ratio of baseline N1/P2 LDAEP for treatment nonresponse, the low LDAEP group had a risk of presenting treatment non-response that was 1.9 times higher. These findings are consistent with previous evidence of an association between a high pretreatment LDAEP in MDD patients, indicating a low central serotonergic activity, and a favorable response to antidepressant agents, SSRIs with a serotonin-enhancing effect [25,26,27,29]. The findings of several studies suggest that a high pretreatment LDAEP can predict a favorable response to short-term (*i.e*., 4 weeks) treatment with an SSRI in MDD patients [25,26,27]. However, the present findings and a recent study by Jaworska *et al.* [28] have observed an association between LDAEP and a treatment response to long-term (at least 12 weeks) maintenance treatment [28]. The present study revealed that higher cortical P2 and N1/P2 LDAEP were associated with treatment responders, while Jaworska and colleagues [28] reported that higher N1 LORETA-LDAEP was associated with responders. Intriguingly, the present study also revealed that higher cortical P2 LDAEP and left P2 sLORETA-LDAEP were associated with treatment remitters.

In addition, some studies have found a high pretreatment LDAEP to be significantly greater in responders to treatment with bupropion or lithium—both of which may affect serotonin activity—than in nonresponders [30,31]. In contrast, other studies have shown that a low pretreatment LDAEP is associated with response to the norepinephrine reuptake inhibitor (NRI) [29,32,33]. Taken together, variables of LDAEP could be associated with response or nonresponse to antidepressant treatment both for short- and long-term durations. It is necessary to verify in future studies whether the pretreatment LDAEP can predict a response to both acute and chronic antidepressant treatment via augmenting serotonin and other neurotransmitters.

Another finding from the present study was the lack of a significant alteration in the LDAEP between baseline and follow-up after maintenance antidepressant therapy. This finding indicates that treatment with serotonin-enhancing agents for more than 12 weeks did not lead to a change in the LDAEP. Previous studies have found no change in the LDAEP following 24 days or 4 weeks of treatment with SSRIs [25,34]. Studies exploring the LDAEP after administering an SSRI in healthy adults have also produced conflicting results: some have found no changes in the LDAEP after a single administration [35,36,37] or after 24-day administration [1] of SSRIs, while other have shown a decrease in the LDAEP after a single trial [38] or after 24-day administration [39] of SSRIs. In addition, some studies have reported the association between altered LDAEP and polymorphisms of the serotonin transporter gene [6,40]. Taken together, these findings suggest that the LDAEP might remain stable in MDD patients before and after SSRI administration.

### 2.4. Study Limitations

This study was subject to several limitations. First, the sample was relatively small; Second, this study did not include a control condition with either a placebo or another non-serotonergic antidepressant drug; Third, the severity of depression was only assessed using the BDI score. It is therefore necessary for future studies with a large sample to assess the cortical and source LDAEP in order to determine which LDAEP variable is a more sensitive marker for predicting a treatment response.

## 3. Experimental Section

### 3.1. Subjects and Study Design

In total, 41 outpatients (7 males and 34 females; 40.2 ± 15.2 years old, mean ± SD) who met the Diagnostic and Statistical Manual of Mental Disorders (DSM)-IV-text revision criteria for MDD were recruited from Ilsan Paik Hospital. Patients were excluded if they had any major mental disorders including anxiety disorder on axis I or II of the DSM-IV, or major medical and neurological disorders. Individuals with hearing impairment were excluded. Patients were either medication-naïve or medication-free for at least eight weeks when entering this study. They all were Korean and of the same ethnicity. Of these, 37% (15/41) of subjects had first-episode MDD, and 63% (26/41) had recurrent-episode MDD. In addition, 24% (10/41) were current smokers. Depressive symptoms were assessed using the Beck Depression Inventory (BDI) at baseline. The pretreatment LDAEP was calculated by measuring the event-related potentials induced by auditory stimuli prior to beginning antidepressants. The MDD patients were treated with the following antidepressants: escitalopram (*n* = 32, 10.0 ± 4.0 mg/day), sertraline (*n* = 7, 78.6 ± 26.7 mg/day), and paroxetine controlled-release (CR) formulation (*n* = 2, 18.8 ± 8.8 mg/day). Among them, 21 patients took hypnotic agents including alprazolam (*n* = 13, 0.25–0.5 mg/day), clonazepam (*n* = 7, 0.25–0.5 mg/day), and zolpidem (*n* = 2, 5–10 mg/day). The post-treatment BDI score and LDAEP were reevaluated in all patients when they had taken antidepressant medications for more than 12 weeks, and their dosage of antidepressants remained the same in the last 4 weeks. The mean duration of antidepressant treatment was 14.1 ± 2.1 weeks (range, 12.9–21.1 weeks).

The SSRI medication could have an influence on results of the LDAEP, and then the baseline LDAEP was measured before antidepressant treatment. Moreover, this study did not enroll patients who had taken any psychotropic agent within 8 weeks before the baseline assessment of LDAEP in our study, except for hypnotic drugs such as alprazolam, clonazepam, and zolpidem.

The subjects were stratified according to their treatment response into responders and nonresponders by comparing their pre- and post-treatment BDI scores; responders were defined as those with a reduction in BDI score of >50% between baseline and follow-up. They were also dichotomized according to their median pretreatment N1/P2 LDAEP into a low or high pretreatment LDAEP group (low- and high-LDAEP groups, respectively). In addition, they were also stratified according to their treatment remission into remitters and nonremitters by comparing their pre- and post-treatment BDI scores; remitters were defined as those with <10s point in the post-treatment Beck Depression Inventory (BDI) score.

The study protocol was approved by the ethics committee of Inje University, Ilsan Paik Hospital, and written informed consent to participate was obtained from all subjects at study entry (IB-3-1105-014, in June 2011).

### 3.2. Electroencephalogram (EEG) Methods

All of the subjects were seated in a comfortable chair in a sound-attenuated room. They were asked to keep their eyes open during the entire testing with their eyes fixated in the pointer on a monitor. The auditory processing consisted of 1000 stimuli with an interstimulus interval between 500 and 900 ms. It was presented in a randomized fashion. Tones of 1000 Hz and 80 ms duration (with 10 ms rise and fall times) were generated by E-Prime software (Psychology Software Tools, Pittsburgh, PA, USA) and presented at five intensities (55, 65, 75, 85, and 95 dB SPL) via headphones (MDR-D777, Sony, Tokyo, Japan). They were presented in a randomized fashion. EEG data were recorded from 64 scalp sites using silver/silver-chloride electrodes according to the international 10–20 system (impedance < 10 kV), using an Auditory Neuroscan NuAmp amplifier (Compumedics USA, El Paso, TX, USA). Data were collected at a sampling rate of 1000 Hz, using a bandpass filter of 0.5–100 Hz. In addition, four electrodes were used to measure both horizontal and vertical electrooculograms.

Data were reanalyzed using Scan 4.3 software with a bandpass filter of 1–30 Hz, and ocular contamination was removed using standard blink-correction algorithms [41]. Event-related potential sweeps with artifacts exceeding 70 mV were rejected at all electrode sites. Rejection rate was <5% per each intensity. For each intensity and for each subject, the N1 peak (negative-most amplitude between 80 and 130 ms after the stimulus) and P2 peak (positive-most peak between 130 and 230 ms after the stimulus) were then determined at the Cz electrode. The peak-to-peak N1/P2 amplitudes were calculated for the five stimulus intensities, and the LDAEP was calculated as the slope of the linear-regression curve.

The Cz electrode was chosen because previous studies have shown this to be a reliable site at which the amplitude is larger than at other electrode sites [15,29,42]. The dipole source analysis for the measurement of LDAEP has been used in some studies [43,44], producing results similar to those obtained when using cortical analysis [42]. Moreover, many LDAEP studies have been conducted based on cortical analysis [45,46,47,48].

### 3.3. Source LDAEP Analysis

On the basis of the averaged, scalp-recorded electric potential, standardized low-resolution brain electromagnetic tomography (sLORETA) was used to estimate current density [49]. The sLORETA technique estimates the standardized source current density by using the realistic 3-shell head model, on the basis of the Montreal Neurological Institute (MNI) 152 template provided by the Brain Imaging Center of the MNI, under the assumption that the activity at any single neuron should be highly synchronized to the activity of its closest neighbors [50]. The solution space is restricted to the cortical gray matter and hippocampus of the head model and partitioned into 6239 voxels at a spatial resolution of 5 mm. Anatomical labels, such as Brodmann areas (BAs), are provided by the use of an appropriate transformation from MNI to Talairach space [51]. The loudness dependence of the source activity (source LDAEP) was determined by calculating current source densities for each subject and each sound pressure level. Two electrodes (M1, M2) were not used in the sLORETA analysis because these electrode locations are not supported by the sLORETA software. The calculated standardized current density was averaged between 60 and 240 ms post-stimulus from the primary auditory cortex (BA41), in accordance with a previous study [26,52]. We calculated the 3 values of current density for the left, right, and averaged data from both hemispheres over the voxels that fall under the primary auditory cortex. The source LDAEP was calculated as the slope of the linear regression of current density of BA41 for the 5 stimulus intensities.

### 3.4. Statistical Analysis

Our data including pre- and post-treatment N1, P2, and N1/P2 LDAEP, and BDI scores showed a Gaussian distribution according to the Kolmogorov-Smimov test (data not shown). The demographic data, clinical variables, and LDAEP values were compared between two groups using Student’s *t*-test, paired *t*-test, and the chi-square. Pearson’s correlation coefficients were calculated to examine the relationships between LDAEP and clinical variables. Sex was considered to be a significant covariate when analyzing the N1, P2, N1/P2 LDAEP values, because the previous studies have reported a significant effect in sex on the LDAEP [37,53]. A general linear model was used while controlling for sex as covariate. All tests were two-tailed, and group differences were tested at the *p* < 0.05 level. The statistical packages used for analysis were SAS 9.3 and SALT 2.5.

## 4. Conclusions

In conclusion, the present findings have revealed an apparent association between a high pretreatment LDAEP and a clinical response to long-term treatment with an antidepressant with serotonin-augmenting effects. The pretreatment LDAEP could thus be a useful marker to predict a potential treatment response among MDD patients. More studies with larger samples should be performed to clarify the relationship between the LDAEP and the treatment response in MDD.
